# Comment on “Examining the effectiveness of ChatGPT responses to frequently asked questions by individuals with postural disorders”

**DOI:** 10.1590/1806-9282.20260381

**Published:** 2026-07-10

**Authors:** Ismail Sivri, Esra Babaoglu, Gamze Gul, Tuncay Colak

**Affiliations:** 1 Kocaeli University, Umuttepe Campus, Faculty of Medicine, Department of Anatomy – Izmit, Turkey.; 2 Zonguldak Bülent Ecevit University, Faculty of Medicine, Department of Anatomy – Zonguldak, Turkey.

Dear Editor,

We read with great interest the recent article by Dursun et al. In this study, the authors aimed to evaluate the quality and readability of ChatGPT 4.0 responses to commonly asked questions about postural disorders. Ten frequently asked questions were selected from a list generated by ChatGPT and were posed to the model without follow-up prompts. The responses were independently assessed by five experts from relevant clinical disciplines using a four-grade evaluation system, and inter-rater reliability was calculated using intraclass correlation coefficients. In addition, readability was analyzed using the Flesch-Kincaid Grade Level. The authors further compared ChatGPT’s responses with peer-reviewed literature to examine consistency with scientific sources. The findings suggested that most responses were rated as excellent or satisfactory, with good inter-rater reliability and an average readability level above the recommended sixth-grade standard for patient education materials^1^.

However, we would like to raise a methodological concern regarding the selection of the questions. The ten questions were generated by ChatGPT itself and then selected by the researchers. They were not obtained from real patient consultations, clinical guidelines, structured surveys, or search engine data such as Google Trends. Therefore, the evaluated questions may not reflect actual patient information needs. This approach limits the study’s real-world relevance and may reduce its validity. For example, in a study, the 20 most frequently searched toothache-related queries identified through Google Trends were submitted to ChatGPT^2^. Furthermore, in another study, 100 real patient records were used to generate the queries, reflecting authentic patient concerns^3^.

Additionally, previous studies have shown that the readability scores of the Large Language Models’ responses can vary significantly when role-based or audience-specific prompts are used. In particular, instructing the model to “explain to a medical layperson” or “explain at a 6th-grade reading level” has been associated with improved readability outcomes^4,5^. In this context, incorporating such prompts may help generate responses that are more accessible and better aligned with patient-centered communication goals.

In conclusion, while this study provides valuable insight into the potential of ChatGPT in patient education on postural disorders, methodological refinements in question selection and prompt design may enhance its real-world applicability. Future research incorporating authentic patient-derived queries and audience-specific prompting strategies could yield more robust, patient-centered evidence.

## Data Availability

The datasets generated and/or analyzed during the current study are available from the corresponding author upon reasonable request.
